# Intersecting trajectories of childhood ADHD, socioeconomic deprivation and distinct multimorbidity patterns in women

**DOI:** 10.1038/s44220-026-00653-1

**Published:** 2026-05-25

**Authors:** Naomi Wilson, Kirstie O’Hare, Helen Minnis, Michael Fleming, Ian Kelleher, Ruchika Gajwani

**Affiliations:** 1 Centre for Developmental Adversity and Resilience, School of Health and Wellbeing, University of Glasgow, Glasgow, UK; 2 National Health Service Greater Glasgow and Clyde, Glasgow, UK; 3 Centre for Clinical Brain Sciences, School of Medicine, University of Edinburgh, Edinburgh, UK; 4 Discipline of Psychiatry and Mental Health, University of New South Wales, Sydney, New South Wales Australia; 5 Department of Public Health, School of Health and Wellbeing, University of Glasgow, Glasgow, UK; 6 Centre for Clinical Brain Sciences, University of Edinburgh, Edinburgh, UK; 7 School of Medicine, University College Dublin, Dublin, Ireland; 8 Faculty of Medicine, University of Oulu, Oulu, Finland; 9 Saint John of God Hospitaller Services Group, Stillorgan, Dublin, Ireland

**Keywords:** Risk factors, Preventive medicine

## Abstract

The long-term consequences of attention deficit hyperactivity disorder (ADHD) in females remain underrecognized both in research and clinical practice. Multimorbidity (two or more long-term conditions) is increasingly viewed as a key outcome yet its prevalence, complexity and sociodemographic determinants are understudied in this group. Here, in this longitudinal cohort study, we examined the combined influence of childhood socioeconomic deprivation and ADHD diagnosis on multimorbidity risk in women in early adulthood (that is, aged 18–32 years) and used latent class analysis to explore clustering patterns. Three findings emerged: females with childhood ADHD had a significantly higher risk of adult multimorbidity; childhood ADHD and socioeconomic deprivation increased this risk both in isolation and synergistically, with 39% of the multimorbidity burden attributable to their interaction; and distinct multimorbidity clusters emerged, with the most adverse marked by psychiatric complexity. These results highlight girls with ADHD from disadvantaged backgrounds as a high-risk group requiring earlier, integrated care.

## Abstract (web summary)

Women diagnosed with attention deficit hyperactivity disorder (ADHD) in childhood have a significantly higher risk of developing physical and mental health conditions (multimorbidity) in early adulthood. ADHD and socioeconomic deprivation independently increase this risk, and in combination have a synergistic effect.

## Main

It is now well recognized that attention deficit hyperactivity disorder (ADHD) in females has been historically underrecognized, underdiagnosed and undertreated^1^. Long viewed as a predominantly male childhood disorder, until the last two decades, female presentations of ADHD—which are often more inattentive and internalized—frequently went undiagnosed^1^. As a result, the long-term consequences of ADHD in girls and women have been largely overlooked in both clinical practice and research^2^. However, growing evidence suggests that relative to males, females with a childhood ADHD diagnosis face a substantially elevated risk of adverse outcomes across the life course, including poorer physical and mental health in adulthood^3–5^. One area of increasing research interest is the risk of multimorbidity, commonly defined as two or more long-term conditions (LTCs) in the same individual. Emerging evidence suggests that this may be particularly prevalent and complex among neurodivergent individuals^6,7^, yet it remains critically understudied in this population generally and among females in particular^8^.

The mechanisms underlying these increased risks are also poorly understood. However, it is clear that ADHD is likely to contribute to poorer health trajectories via multiple interrelated pathways^8^. For example, biological pathways, such as dysregulated stress responses and inflammation, are likely to interact with behavioral pathways, such as impulsivity-related risk behaviors, to influence an individual’s overall risk^8^. Crucially, ADHD rarely occurs in isolation from structural influences and inequalities^9^. Among these, socioeconomic disadvantage is the factor most consistently and strongly associated with both childhood ADHD diagnoses^10^ and adverse adult health outcomes^11^. While many studies therefore aim to disentangle the effects of ADHD and socioeconomic status (SES) to determine their relative influence, in clinical reality, they frequently co-occur^12^. As such, there is a pressing need to examine not only their individual contributions but also their combined and potentially interacting effects^13^. Nevertheless, current literature rarely examines these factors in combination, and the additive or synergistic effects of socioeconomic disadvantage and ADHD on long-term health outcomes have so far not been systematically explored in females^13^.

Moreover, while most studies treat multimorbidity as a homogeneous end point, it is now well recognized that LTCs tend to cluster into distinct, nonrandom groupings with considerable variation in the severity of outcomes they confer^14^. For example, musculoskeletal conditions commonly co-occur with depression, while renal conditions co-occur with cardiovascular disease^15^—each cluster potentially reflecting different etiological pathways and impacting on different functional outcomes^16^. These clusters are also associated with differing patterns of healthcare utilization. For instance, individuals with co-occurring mental and physical health conditions have been associated with increased secondary care use and potentially preventable hospital admissions^17^, while the clustering of autoimmune conditions—more commonly observed in women—is often linked to higher primary care attendance^18,19^. Understanding multimorbidity clustering and the associated patterns of healthcare use is thus crucial for helping healthcare services to better meet the needs of those with multiple conditions, through designing tailored management strategies and developing more precise public health responses. In females with ADHD—a population that often faces unique challenges such as later or missed diagnosis, internalizing symptom profiles and heightened exposure to social and educational disadvantage—the identification of specific multimorbidity clusters may be particularly valuable^5,20^. Through revealing distinct health trajectories, and thus the potential underlying mechanisms of risk, this could inform more targeted, sex-sensitive interventions. However, so far, no study has investigated such patterns in this group.

To address these gaps, here, we first examine the interaction between socioeconomic deprivation and ADHD in shaping the multimorbidity risk of adult women (aged between 18 and 32 years) with a childhood diagnosis of ADHD (that is, with a diagnosis made at any point before the age of 18 years) and second use latent class analysis to explore the consequent patterns of multimorbidity in this population. In doing so, we aim to address three key questions:

1. How does socioeconomic deprivation interact with ADHD to influence the risk of future multimorbidity in females—that is, is there an additive or synergistic effect?
2. What are the distinct clusters of multimorbidity observed in this population?
3. How do these multimorbidity clusters influence healthcare use (specifically the number of hospital attendances and duration of hospital admissions)?

## Results

### Sociodemographic characteristics, prevalence and risk of multimorbidity

**Fig. 1:**
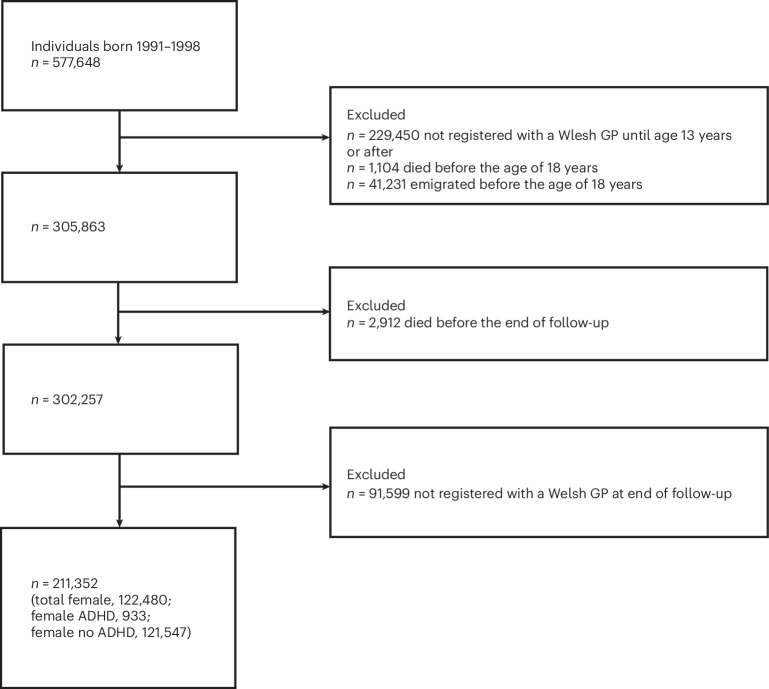
Cohort flow diagram. Flow diagram showing inclusion and exclusion of individuals born in Wales between 1991 and 1998, resulting in the final analytical sample.

The participant flow through the study is shown in Fig. 1. The sociodemographic characteristics of the original and matched sample are presented in Table 1.

**Table 1:** Sociodemographic characteristics of original and matched sample

Total (*n*)	Before matching			After matching		
	ADHD	No ADHD	Standardized bias	ADHD	No ADHD	Standardized bias
	933	121,547		933	9,330	
Age (years (mean, s.d.))						
Study entry	0.8 (2.3)	0.6 (2.1)	−0.17	0.8 (2.3)	0.6 (2.1)	−0.01
ADHD diagnosis	9.4 (4.2)	–		9.4 (4.2)	–	
Study exit	28.1 (2.3)	28.5 (2.3)		28.1 (2.3)	28.1 (2.3)	
Intellectual disability (*n*(%))	103 (11.0)	1,192 (1.0)	0.32	103 (11.0)	1,030 (11.0)	<0.01
Low birth weight (*n*(%))	145 (15.5)	12,607 (10.4)	0.14	145 (15.5)	1,443 (15.5)	<0.01
Urban residency at birth (*n*(%))	668 (71.6)	85,504 (70.3)	0.03	668 (71.6)	6,627 (71.0)	0.01
Out-of-home care (*n* (%))	144 (15.4)	2,036 (1.7)	–	144 (15.4)	363 (3.9)	
WIMD quintile at baseline						
1	372 (39.9)	34,108 (28.1)	0.24	372 (39.9)	3,728 (39.9)	<0.01
5	93 (10.0)	17,235 (14.2)		93 (10.0)	1,086 (11.6)	
Multimorbidity^a^						
Multimorbidity	879	102,099	–	879	8,139	–
No multimorbidity	54	19,448	–	54	1,191	–

^a^Multimorbidity defined as ≥2 long-term conditions.

Before propensity score matching, the most notable differences between the ADHD and non-ADHD groups were in the prevalence of intellectual disability, low birth weight, socioeconomic deprivation (as assessed by Welsh Index of Multiple Deprivation (WIMD) quintile) and periods of out-of-home care. After matching, the differences between those with and without ADHD were substantially reduced in all selected covariates, and the standardized bias before and after matching suggested that the procedure was successful in creating a comparable control group (that is, standardized bias <0.1).

In both groups, the mean age of the participants at the end of follow-up was 28.1 (s.d. 2.3) years, with a range of 18–32 years and an average of 10.1 (s.d. 2.3) years of follow-up. Compared with those without ADHD in both the unmatched (unadjusted odds ratio (OR) 3.10, 95% confidence interval (CI) 2.4 to 4.1) and matched sample (adjusted OR 2.38, 95% CI 1.8 to 3.2), those with a childhood diagnosis of ADHD were found to be at a significantly increased risk of multimorbidity.

### Additive effects of childhood ADHD diagnosis and socioeconomic disadvantage

**Fig. 2:**
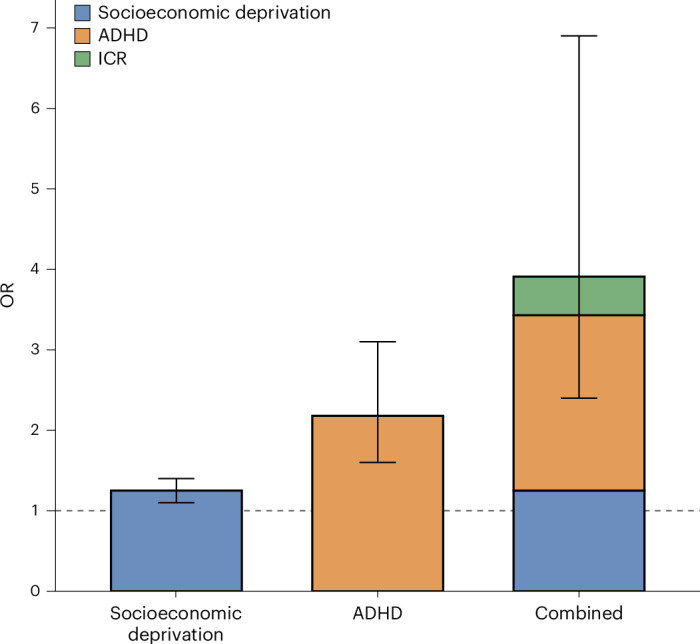
Odds ratios for multimorbidity by ADHD, socioeconomic deprivation and combined exposure. The bars represent estimated effects, and the error bars indicate 95% CIs. The stacked bar represents the combined effect of deprivation and ADHD. The horizontal reference line at 1 indicates the null value for ORs. The unit of analysis is the individual participant. Sample sizes were as follows: ADHD-only group (*n* = 561), deprived-only group (*n* = 3,728) and combined group (*n* = 372). Estimates are based on observational population-level data.

The findings of an additive interaction analysis indicated a clear additive interaction between childhood ADHD and socioeconomic disadvantage in increasing the risk of multimorbidity. Individuals with ADHD who were also in the most deprived socioeconomic quintile in childhood had the highest odds of multimorbidity (OR 3.91, 95% CI 2.4 to 6.9), considerably greater than those with either risk factor alone (Fig. 2 and Table 2). The interaction contrast ratio (ICR) was 1.48 (95% CI −0.7 to 3.4), suggesting a probable synergistic effect. Importantly, the attributable proportion (AP) of 39% indicated that a substantial portion of the risk in the co-exposed group may be due to the interaction between ADHD and deprivation.

**Table 2:** Additive effects of ADHD and deprivation exposure on risk of multimorbidity

	Most deprived = 0		Most deprived = 1		ICR (95% CI)	AP
	MM/no MM	OR (95% CI)	MM/no MM	OR (95% CI)		
ADHD = 0	4,829/773	(Ref.)	3,310/418	1.25 (1.1 to 1.4)	1.48 (−0.7 to 3.4)	39%
ADHD = 1	522/39	2.18 (1.6 to 3.1)	357/15	3.91 (2.4 to 6.9)		

MM, multimorbidity.

### Multimorbidity clusters and associated outcomes

Among those with multimorbidity (that is more than two LTCs), Bayesian information criterion values indicated an optimum number of three clusters in those without a childhood ADHD diagnosis and two clusters in those with a childhood ADHD diagnosis (Supplementary Fig. 1a,b and Supplementary Table 2).

**Fig. 3:**
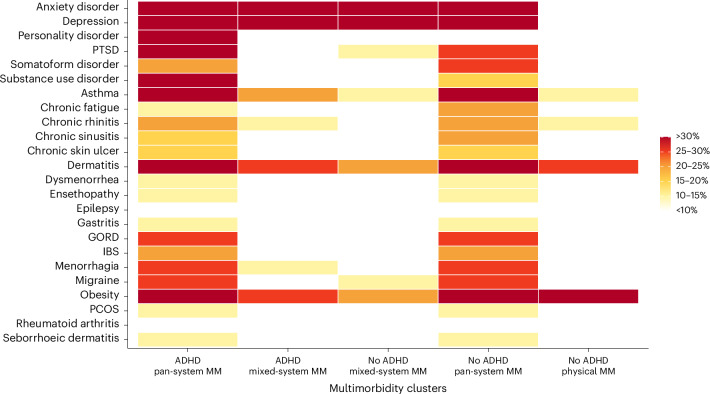
Prevalence of top 15 overlapping LTCs included in each multimorbidity cluster. Colors represent prevalence categories based on the proportion of individuals within each multimorbidity cluster diagnosed with each long-term condition. Prevalence estimates are derived from an observational cohort of *n* = 10,263 individuals. The unit of analysis is the individual participant. GORD, gastroesophageal reflux disease; IBS, irritable bowel syndrome; PCOS, polycystic ovary syndrome; PTSD, posttraumatic stress disorder.

Latent class analysis (LCA) subsequently assigned individuals from both the ADHD and non-ADHD group into each of these five clusters, and the probabilities of an individual having each included LTC was extracted, as was the actual number (and percentage) of individuals with a diagnosis of the LTC. Although we included all 76 LTCs in the latent class models, here we will discuss only the 15 most prevalent conditions in each cluster for clarity. Prevalence rates for each of the most prevalent 15 conditions in each cluster were consequently mapped for comparison of similarities and differences across ADHD and non-ADHD groups. These are shown figuratively in Fig. 3, while exact prevalence rates and cluster-specific probabilities (that is, the probability of an individual in each cluster having a specific LTC) for the top 15 LTCs in each cluster are presented in Supplementary Table 3.

In characterizing the multimorbidity clusters, we observed three distinct patterns based on condition frequency and spread across body systems. To aid interpretation and clinical relevance, we chose to name each cluster on the basis of these patterns. The first, which we termed ‘physical multimorbidity’, was defined by LTCs concentrated within physical health domains only (for example, respiratory, metabolic or gastrointestinal). This cluster was only observed in those with no childhood ADHD diagnosis. The second pattern, which we termed ‘mixed-system multimorbidity’, encompassed both physical LTCs and common psychiatric conditions (that is, anxiety and depression), indicating broader cross-domain health needs. Finally, the most extensive pattern, ‘pan-system multimorbidity’, reflected both a higher burden of LTCs spanning the majority of physical body systems and mental health domains, representing the most wide-reaching multimorbidity profile. Both mixed-system and pan-system multimorbidity patterns were present among those with and without a childhood ADHD diagnosis. However, when comparing these clusters between groups, a higher prevalence of multiple major psychiatric disorders was observed in those with a childhood diagnosis of ADHD compared with those with no such diagnosis, indicating higher psychiatric load and complexity. Specifically, borderline personality disorder was uniquely concentrated in this cluster for those with a childhood ADHD diagnosis, where its prevalence was high, while appearing absent from all other clusters; moreover, posttraumatic stress disorder was also more common in this group (Fig. 3).

The sociodemographic characteristics and health outcomes (that is, mean number of LTCs, hospital admissions and total days in hospital) associated with each cluster are presented in Table 3. Across all multimorbidity clusters, a larger proportion of participants resided in the most deprived WIMD quintile at birth. Overall, the cluster with the highest mean number of hospital admissions, number of days spent in hospital and LTCs was the ADHD pan-system multimorbidity cluster, closely followed by the non-ADHD pan-system multimorbidity cluster (Table 3).

**Table 3:** Sociodemographic characteristics and associated outcomes of multimorbidity clusters in ADHD and non-ADHD groups

	Non-ADHD group			ADHD group	
	Pan-system MM (*n* = 1,632)	Mixed-system MM (*n* = 2,780)	Physical MM (*n* = 3,727)	Pan-system MM (*n* = 308)	Mixed-system MM (*n* = 571)
Sociodemographic characteristics (*n* (%))					
Most deprived	709 (43.4)	1,280 (46.04)	1,321 (35.4)	136 (44.2)	221 (38.7)
Least deprived	159 (9.7)	269 (9.7)	474 (12.7)	25 (8.1)	61 (10.7)
Associated outcomes (mean (s.d.))					
Mean number of LTCs	10.3 (2.7)	5.0 (1.7)	4.0 (1.7)	11 (3.1)	5.0 (1.9)
Mean number of hospital attendances	8.2 (9.5)	4.8 (5.1)	4.1 (6.2)	10.0 (9.7)	5.5 (5.2)
Mean number of days in hospital	18.7 (81.3)	6.4 (20.8)	5.3 (24.3)	25.5 (77.5)	10.3 (44.5)

## Discussion

This study aimed to examine how childhood ADHD diagnosis and socioeconomic deprivation interact to shape the risk of multimorbidity in females and to identify distinct multimorbidity patterns within this population using LCA. Our key findings were threefold. First, females with a childhood ADHD diagnosis were at a significantly increased risk of adult multimorbidity compared with matched controls. Second, while both ADHD and childhood socioeconomic deprivation were individually associated with increased risk, their co-occurrence resulted in markedly higher odds of multimorbidity, suggesting a possible additive interaction. Finally, distinct clusters of multimorbidity were identified, with the most complex and adverse cluster (pan-system multimorbidity) among individuals with ADHD being characterized by a higher burden of major psychiatric disorders.

These findings build on a growing body of research indicating that ADHD in females is associated with a wide range of long-term health difficulties^21^, extending beyond the core symptoms of the disorder^2^. Existing literature has highlighted cross-sectional associations between both common mental health disorders (that is, anxiety and depression)^22^ and selected physical LTCs in those with ADHD^23^. However, few studies have examined how LTCs co-occur and cluster in adulthood or how structural inequalities may amplify these risks longitudinally in females specifically^24^. By incorporating both interaction analysis and latent class modeling, this study extends previous work by showing not only the elevated prevalence of multimorbidity in this group but also the complexity and heterogeneity of its presentation.

The observed additive interaction between ADHD and socioeconomic deprivation aligns with broader public health research demonstrating that individuals facing multiple vulnerabilities are at higher cumulative risk for poor health outcomes^25^. However, in both research and clinical practice, the social and biological determinants of health largely continue to be considered separately^26,27^. This ignores their dynamic interplay and may lead to incomplete or misleading conclusions about disease causation and population health trends. Our findings demonstrate how these factors jointly influence risk trajectories across the life course and offer new insights by quantifying this interaction specifically in a female ADHD population—a group typically underrepresented in existing health research generally^28^. In this female sample, the ICR suggested a probable synergistic effect between childhood ADHD diagnosis and socioeconomic deprivation, with the AP indicating that up to 39% of the multimorbidity burden in those with both may be due to the interaction between them itself. This highlights the importance of considering how structural disadvantage may interact with neurodevelopmental conditions specifically to shape health trajectories, particularly among women.

In line with existing studies showing that multimorbidity does not occur at random^29^, our LCA also revealed distinct patterns of co-occurring LTCs among this population. Notably, the most complex cluster among females with ADHD, pan-system multimorbidity, included a particularly high psychiatric burden—more so than the corresponding pan-system multimorbidity cluster in females without ADHD—suggesting the presence of a unique and more severe profile. These findings support calls for a shift away from treating multimorbidity as a homogeneous outcome^30^ and underscore the need for targeted, sex-sensitive care pathways that address specific clinical constellations^31^.

Overall, this study’s findings are strengthened through its use of a large, population-based dataset with linked primary and secondary care records, providing rich longitudinal data on health trajectories. The application of both propensity score matching and LCA also enhances causal inference and permits nuanced subgroup identification. However, while interpreting our results, there are also limitations which should be noted. Clinical ADHD diagnoses are known to be influenced by a range of factors beyond symptom severity, including sex biases and differential access to healthcare^1^. It is therefore likely that some individuals within the ‘control’ group had an undiagnosed ADHD profile. This potential misclassification may have biased estimates toward the null, underestimating the true magnitude of associations. This is also true of all LTCs included in our multimorbidity outcome, as within routinely collected electronic health records, the absence of diagnostic codes may reflect healthcare utilization and recording practices rather than true absence of disease, which could lead to underascertainment of some conditions and potentially conservative estimates of associations.

Our measure of socioeconomic deprivation similarly requires consideration, as this was derived from an area-based, as opposed to an individual-based, measure, and so it is likely some individuals may have been misclassified. In addition, there is currently no universally accepted definition of multimorbidity in young adult populations^32^. Our finding that a large proportion of those in both groups met our definition of ‘multimorbidity’ must therefore be considered in light of the fact that we purposely adopted a broad and inclusive definition. This decision was made because it is likely that multimorbidity in young adults encompasses a diverse range of LTCs and such an approach is also appropriate for the use of cluster analysis, which aims to detect underlying patterns of co-occurrence across a wide spectrum of conditions. However, this also means that we are likely to have captured individuals with less severe or functionally impairing conditions. Nonetheless, the definition used had been validated through consensus among experts in a previous study and permitted a more granular and inclusive analysis of multimorbidity than has been typical in similar studies. Finally, a key consideration is that the sample was matched on socioeconomic deprivation, which was subsequently examined as an effect modifier. While this could be perceived as a methodological limitation, in the current study it was a deliberate design decision, chosen to enhance internal validity and reduce confounding, and as it has previously been widely used in other similar studies^33,34^. Matching on socioeconomic deprivation ensured comparability between ADHD and non-ADHD groups across a key structural determinant of health. Importantly, the interaction between ADHD and socioeconomic deprivation was assessed within this covariate-balanced sample, prioritizing robustness and internal consistency. As matching reduces the range of deprivation and excludes individuals at the extremes, the observed interaction is probably a conservative estimate. In the general population—where greater socioeconomic variability exists—the combined impact of ADHD and deprivation may be even stronger.

## Conclusion

This study provides new insights into the complex interplay between ADHD, socioeconomic disadvantage and adult multimorbidity in females. Clinically, the findings suggest that girls with ADHD, particularly those from disadvantaged backgrounds, should be considered a high-risk group for long-term health complications and may benefit from earlier, more integrated care approaches. From a policy perspective, the results highlight the need for public health strategies that address both neurodevelopmental and social determinants of health together, rather than in isolation. Several related questions also remain that warrant further investigation. Comparative research is needed to determine whether the observed multimorbidity risk and clustering of LTCs in women with a childhood ADHD diagnosis are similar to, or distinct from, those seen in women with other childhood neurodevelopmental diagnoses, as evidence in this area remains limited. As this study was conducted using a single, population-based dataset from one national context, replication in other populations and healthcare systems will also be important to assess the generalizability of these findings, particularly given known variation in diagnostic practices and service provision. In addition, intergenerational factors such as parental ADHD have previously been shown to influence socioeconomic circumstances and subsequent health trajectories^35^, but this could not be examined in the present dataset and represents an important avenue for future work. The role of ADHD treatment, including stimulant medication use, in shaping long-term multimorbidity patterns and healthcare utilization also requires dedicated study, as treatment effects may meaningfully modify health outcomes across the life course. Finally, future research should explore how sex-sensitive early interventions can disrupt the compounding effects of ADHD and social disadvantage on health outcomes across the life course. Addressing these gaps is essential to developing equitable, life-course approaches that reduce long-term health inequalities in neurodivergent women.

## Methods

### Data access and ethics

Access to the datasets used to conduct this study was granted following review and approval by the Secure Anonymised Information Linkage (SAIL) Information Governance Review Panel (IGRP; number 1635), which includes representation from data providers and independent members. As this study used anonymized secondary data, individual participant consent was not required.

### Study design

This observational cohort study is reported as per the Strengthening the Reporting of Observational Studies in Epidemiology (STROBE) guidelines^36^.

### Data sources

Participants were identified from linked data hosted in the SAIL databank, a national data repository for Wales. SAIL enables the secure linkage of individual-level data across multiple datasets using anonymized linkage fields, generated through a privacy-protecting split-file process, allowing records belonging to the same individual to be combined without the use of identifiable information. Linkage quality is routinely assessed.

Incidences of exposure, outcome and confounders were identified from a range of datasets hosted within the SAIL databank^37–42^, specifically: the Welsh Demographic Service Dataset; the National Community Child Health Database (NCCHD); the Welsh Longitudinal General Practice (WLGP) Database containing electronic health records from ~80% of general practitioner practices in Wales, covering ~83% of the population; and the Patient Episode Database for Wales (PEDW). These datasets were linked at the individual level within the SAIL trusted research environment and analyzed using de-identified, longitudinal records.

### Missing data

As all variables were derived from routinely collected health data, the absence of a clinical or administrative code was interpreted as absence of the condition or characteristic, and all individuals remained in the analysis.

### Cohort

Participants were included if they were female, born between 1991 and 1998 (inclusive), were registered with a Welsh general practice both before the age of 13 years and had not died or emigrated before the end of follow-up (*n* = 122,480). A participant flow diagram is available in the Supplementary Information (Supplementary Fig. 1).

### Exposure

Incidences of ADHD diagnosed before the age of 18 years were ascertained through both primary (WLGP) and secondary (PEDW) care records, using a list of validated International Classification of Diseases, Tenth Revision (ICD-10), and National Health Service Read Codes^43^ presented in Supplementary Tables 4 and 5.

### Outcomes

#### Multimorbidity

Multimorbidity was broadly defined as the coexistence of two or more LTCs according to the Academy of Medical Sciences definition^44^. The specific LTCs included were determined through refining the multimorbidity index initially developed by Eto and colleagues (2023) when estimating the prevalence of multimorbidity among 16–39-year-olds in England^45^. Specifically, 76 LTCs found to have a rounded prevalence of 2% or greater in the total population in this study were ultimately included (Supplementary Table 1). Code lists for each of the selected conditions were then developed through combining validated ICD-10 code lists from the original study^46^ and validated Read code lists available in the concept library provided by the SAIL databank. Diagnoses of these conditions made after the age of 18 years were then extracted from both general practitioner (GP) and inpatient hospital records in the SAIL databank using the first relevant code recorded after the age of 18 years. If a condition was never recorded, it was considered absent. Participants were then categorized into those with and without multimorbidity (that is, ≥2 LTCs = 1; <2 LTCs = 0).

Healthcare use:

- Number of LTCs: The number of LTCs diagnosed for each participant was additionally included as a count variable.
- Hospital attendances and length of hospital stay: The number of hospital attendances and length of admissions (treated as a continuous variable) were obtained from the PEDW. All inpatient episodes over the age of 18 years were extracted, capturing admissions for all causes across all hospital specialties.

### Confounders

Confounders included:

1. Birth year: identified from the WDSD
2. Birth sex: identified from the WDSD and categorized as male or female
3. SES at birth: measured using the 2014 version of the WIMD and identified from the individual’s earliest available record in the WDSD. A binary indicator was created, which categorized participants into those belonging either to the most deprived quintile of the WIMD or the other four quintiles combined
4. Urban environment at birth: indexed using the 2011 rural–urban classification of each individual’s earliest available area code record in the WDSD
5. Low birth weight: classified as <2,500 g and extracted from the NCCHD
6. Lifetime intellectual disability (ID): identified through primary care (WLGP) and secondary care records (PEDW) using a list of previously validated Read and ICD-10 codes^47^

Ethnicity information is known to be incompletely and inconsistently recorded in routinely collected electronic health records. In this study, owing to incomplete recording, the number of individuals from minority ethnic groups was insufficient to permit reporting of ethnicity categories without risking disclosure; therefore, ethnicity was not included in the analyses or demographic tables.

Birth year was included as a continuous variable; all other confounders were included as binary variables.

### Statistical analysis

To address potential confounding, a propensity score-matched sample was first constructed in which participants with ADHD were matched to participants without ADHD in a 1:10 ratio on the basis of all measured confounders. Propensity score matching aims to achieve covariate balance between exposure and control groups in observational data, thereby closely mimicking a randomized design and allowing for straightforward estimation of prevalence rates after accounting for confounding^48^. Unlike traditional covariate adjustment, which models confounding within the outcome regression and may be sensitive to model specification, matching on the propensity score separates confounding control from outcome analysis, reducing model dependence and improving interpretability of treatment effects^49^. Propensity scores were estimated using logistic regression, and nearest-neighbor matching without replacement was implemented using the MatchIt package in R. Using this matched sample, the prevalence and odds of multimorbidity among individuals with ADHD, compared with those without ADHD, were calculated using logistic regression. All participants entered the analysis at age 18 years (regardless of timing of ADHD diagnosis) and were classified according to whether multimorbidity occurred at any time during the follow-up period (that is, up to administrative censoring at the end of the study period (November 2023)), excluding those who had died or emigrated, giving participants a maximum age of 32 years at the study end point.

Next, the individual and combined effects of childhood ADHD diagnosis and poverty on the risk of multimorbidity was assessed in an additive interaction analysis. To achieve this, we followed the methodology recommended by Knoll and VanderWeele^50^. Specifically, individuals were categorized into four mutually exclusive exposure groups: (1) neither ADHD nor low SES (reference group), (2) ADHD only, (3) low SES only and (4) both ADHD and low SES. ORs for the risk of multimorbidity in each group were then calculated, and the presence of an additive interaction was evaluated using the ICR, which quantifies whether the combined effect of both exposures exceeds the sum of their individual effects, and was calculated according to the formula below:

$$\begin{array}{l}{\mathrm{OR}}_{{\mathrm{ExposureAB}}\,({\rm{i}}.{\rm{e}}.{\mathrm{ADHD}}\,{\mathrm{and}}\,{\mathrm{poverty}})}-{\mathrm{OR}}_{{\mathrm{ExposureA}}\,({\rm{i}}.{\rm{e}}.{\mathrm{ADHD}})}\\-{\mathrm{OR}}_{{\mathrm{ExposureB}}\,({\rm{i}}.{\rm{e}}.{\mathrm{poverty}})}+1\,=\,{\mathrm{ICR}}\end{array}$$

An ICR greater than zero suggests a synergistic (positive) interaction on the additive scale, indicating that the joint effect of ADHD and poverty is greater than expected on the basis of their separate contributions. CIs for the ICR were then calculated using the delta method^51^. This approach was chosen, as there is now general consensus in the epidemiological community that measuring interaction on the additive scale is the most appropriate for assessing the public health importance of said interactions.

Finally, LCA was used to identify distinct clusters of LTCs among individuals with multimorbidity, separately in those with and without a childhood ADHD diagnosis. LCA models were estimated separately because our aim was to characterize how LTCs cluster within each group, rather than to assume that the same pattern of condition co-occurrence applies across both populations. A pooled model would require this shared structure assumption, with ADHD influencing only the probability of belonging to each cluster. Estimating models separately allowed group-specific multimorbidity patterns to emerge without imposing this constraint. Our aim was also to uncover underlying patterns in the co-occurrence of LTCs that may reflect clinically meaningful subgroups within each population. To achieve this, optimal LCA model selection (that is, number of clusters) was first determined in each group through model parsimony (using the Bayesian information criterion)^52^. LCA using this optimal number of clusters for each sample was then conducted using the poLCA package in R. LCA is a person-centered mixture modeling approach, which can be used to identify subpopulations within a sample on the basis of individuals’ patterns of responses to observed variables, in this case the presence or absence of an LTC, across multiple binary LTC indicators^53^. This is achieved through estimating posterior probabilities for each individual—that is, the probability of belonging to each subgroup given an individuals’ specific combination of LTCs. These posterior probabilities are then used to assign each individual to the class for which they had the highest probability of membership, a common approach that facilitates interpretation and downstream analysis^54^. The mean number of LTCs, hospital attendances and days in hospital were then calculated for each cluster. Lastly, to further interpret the identified clusters, we then extracted the class-specific conditional probabilities for each LTC, meaning the probability that an LTC was present within each class. The 15 most prevalent LTCs in each class were then identified on the basis of these probabilities and were used to characterize the unique clinical profiles of each subgroup.

All analyses took place between December 2024 and July 2025. SQL Db2 was used to interrogate data in the SAIL Databank, and analyses were conducted using R version (4.3.1).

### Reporting summary

Further information on research design is available in the Nature Portfolio Reporting Summary linked to this article.

## Supplementary information

Supplementary Information Supplementary Tables 1–5 and Fig. 1a,b.

Reporting Summary

## Data Availability

The data that support the findings of this study are available from the Secure Anonymised Information Linkage (SAIL) Databank. These data are not publicly available owing to governance and privacy restrictions but can be accessed by bona fide researchers via application to the SAIL Databank’s independent Information Governance Review Panel (IGRP). Access requires approval of a research proposal and must be conducted within the SAIL secure Trusted Research Environment. Further details regarding the application process are available at https://www.saildatabank.com.

## Code availability

The analyses presented in this study were implemented in R (4.3.1; R Foundation for Statistical Computing). Propensity score matching was performed using the MatchIt package (4.7.2) available via CRAN at https://cran.r-project.org/web/packages/MatchIt/index.html. Latent class analysis was conducted with the poLCA package (1.6.0.1) available via CRAN at https://cran.r-project.org/web/packages/poLCA/index.html. Standard logistic regression models were estimated using the glm() function in the base stats package of R and the interaction contrast ratio was calculated as per the formula given.
