# Supplementary material for: Intersecting trajectories of childhood ADHD, socioeconomic deprivation and distinct multimorbidity patterns in women
Source: Nat Ment Health. 2026 May 25;4(8):1239–47. doi: 10.1038/s44220-026-00653-1 (PMC13441990; doi:10.1038/s44220-026-00653-1)
Supplement: Supplementary file 1 [file 44220_2026_653_MOESM1_ESM.pdf]

nature mental health

Supplementary information

https://doi.org/10.1038/s44220-026-00653-1

Intersecting trajectories of childhood ADHD, socioeconomic deprivation and distinct multimorbidity patterns in women

In the format provided by the authors and unedited

Supplementary Material

- sTable 1. Included Physical and Mental LTCs
- sTable 2. BIC Values for Different Latent Class Models in ADHD and Non-ADHD Group
- sFigure 1A. No-ADHD Group BIC Values
- sFigure 1B. No-ADHD Group BIC Values
- sTable 3. Probabilities of LTCs, and Numbers and Percentages of Individuals with those Conditions in Each Cluster
- sTable 4. ADHD READ Codes
- sTable 5. ADHD ICD-10 Codes

sTable 1. Included Physical and Mental LTCs

**Included Physical Long-Term Conditions**	
**Neoplasms**	**Musculoskeletal Conditions**
Cervical carcinoma in situ	Enthesopathies and synovial disorders
Primary malignancy breast	Fibromyalgia
Primary malignancy other skin and subcutaneous tissue	Gout
Primary malignancy others	Intervertebral disc disorders
**Haematological Conditions**	Osteoarthritis
Iron deficiency with and without anaemia	Osteoporosis
Other anaemias	Rheumatoid arthritis
Vitamin B12 deficiency with and without anaemia	Spondylosis
**Endocrine/Metabolic Conditions**	**Genitourinary Conditions**
Obesity	Chronic kidney disease
Polycystic ovarian syndrome	Dysmenorrhoea
Thyroid disease	Endometriosis
Type 2 diabetes	Erectile dysfunction
**Neurological Conditions**	Female genital prolapse
Chronic fatigue	Female infertility
Epilepsy	Male infertility
Painful conditions	Menorrhagia and polymenorrhoea
Sleep apnoea	Urinary incontinence
**Eye Conditions**	Urolithiasis
Diabetic eye disease	**Gastrointestinal Conditions**
Visual impairment and blindness	Constipation
**Ear Conditions**	Cholelithiasis
Hearing loss	Diverticular disease of intestine
Migraine	Gastritis and duodenitis
Tinnitus	Gastro-oesophageal reflux disease
**Cardiovascular Conditions**	Irritable bowel syndrome
Atrial fibrillation and flutter	Non-alcoholic fatty liver disease and steatohepatitis
Cerebrovascular disease	Oesophagitis and oesophageal ulcer
Coronary heart disease	Peptic ulcer disease
Heart failure	**Dermatological Conditions**
Heart valve disease non-rheumatic	Atopic Dermatitis
Hypertension	Chronic ulcer of the skin
Peripheral venous and lymphatic disease	Psoriasis
Venous thromboembolism	Rosacea
**Respiratory Conditions**	Seborrheic dermatitis
Allergic and chronic rhinitis	Urticaria
Asthma	
Chronic obstructive pulmonary disease	
Chronic sinusitis	
**Included Mental Long-Term Conditions**	
**Common Mental Disorders**	**Severe and Enduring Mental Illnesses**
Anxiety	Bipolar Affective Disorder
Depression	Personality Disorders
Eating Disorders	Psychotic Disorders
Obsessive Compulsive Disorder	**Addiction Disorders**
PTSD	Alcohol Use Disorder
Somatoform Disorders	Drug Use Disorder

sTable 2. BIC Values for Different Latent Class Models in ADHD and Non-ADHD Group

Number of Classes	BIC Values	
	No ADHD Group	ADHD Group
1	118290.1	17602.50
2	116636.4	17531.84
3	115252.9	18285.41
4	115481.1	18094.72
5	115507.7	18328.45
6	116475.0	18776.61
7	116465.7	19050.20
8	116623.5	19349.38
9	117317.3	19664.85
10	118743.2	20051.18

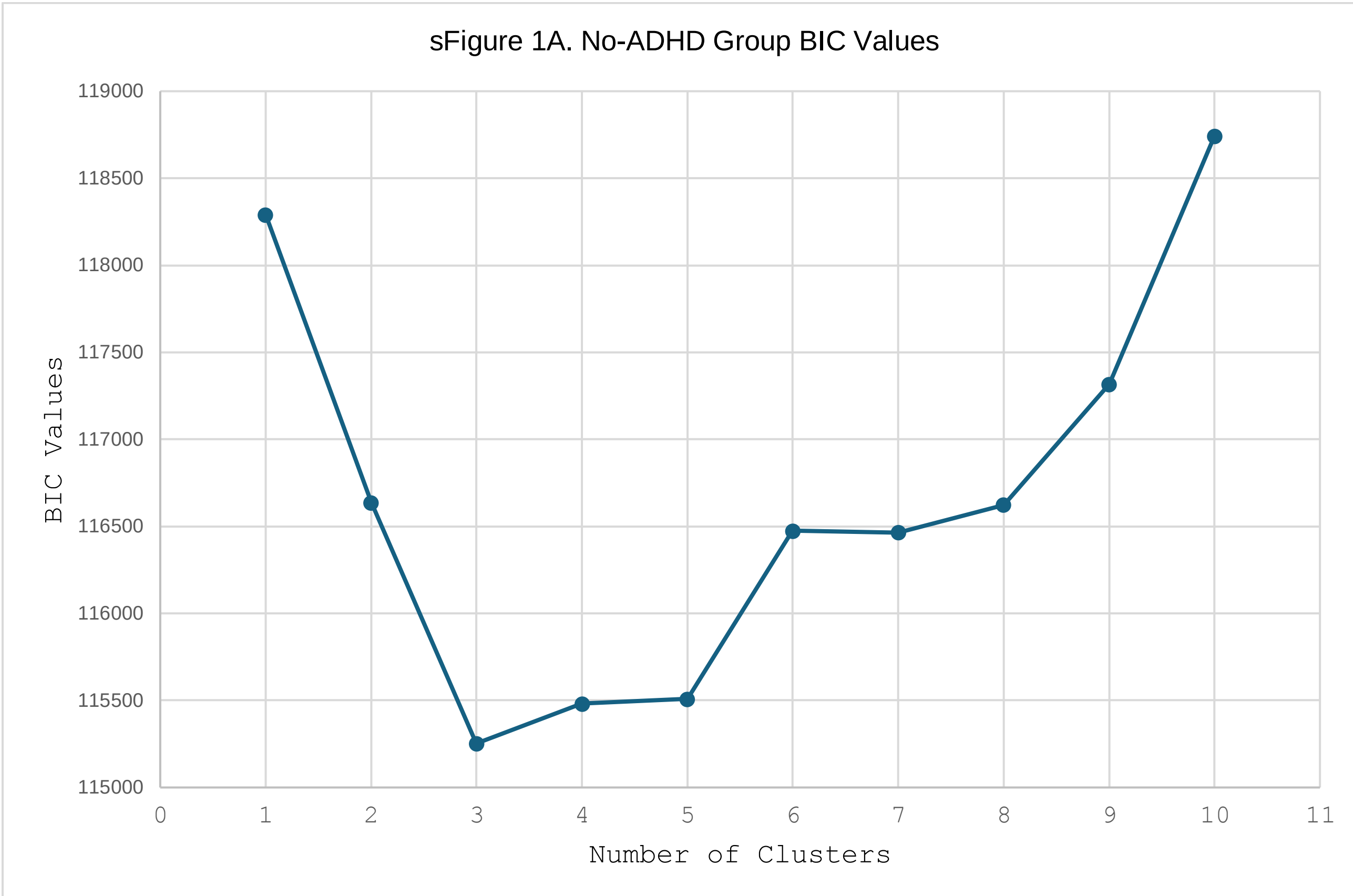
sFigure 1A. No-ADHD Group BIC Values
119000
118500
118000
117500
117000
116500
116000
115500
115000
BIC Values
0
1
2
3
4
5
6
7
8
9
10
11
Number of Clusters

sFigure 1B. ADHD Group BIC Values

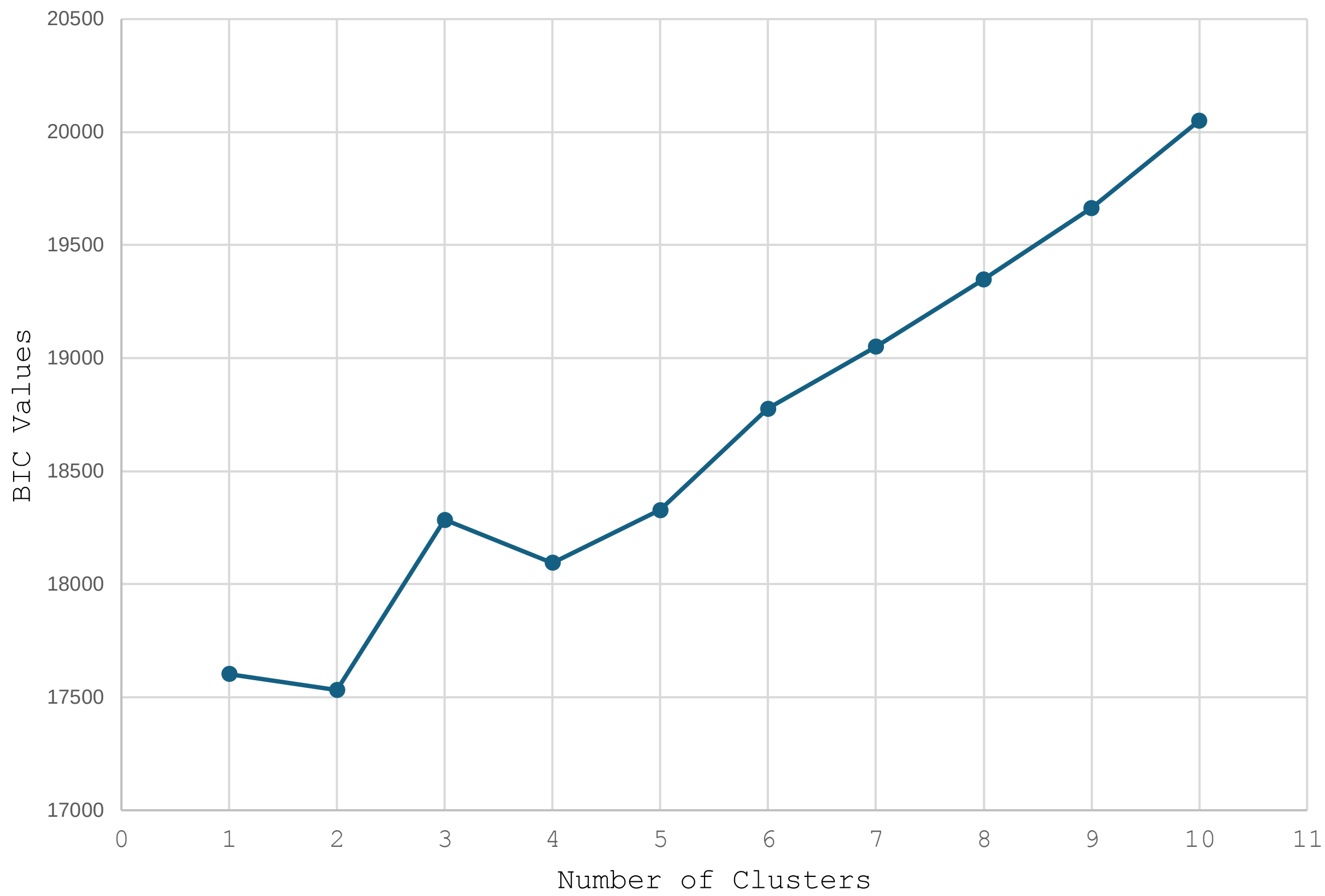

sTable 3. Probabilities of LTCs, and Numbers and Percentages of Individuals with those Conditions in Each Cluster

Cluster																			
**ADHD 1**				**ADHD 2**				**No ADHD 1**				**No ADHD 2**				**No ADHD 3**			
**LTC**	**Prob**	**n**	**%**	**LTC**	**Prob**	**n**	**%**	**LTC**	**Prob**	**n**	**%**	**LTC**	**Prob**	**n**	**%**	**LTC**	**Prob**	**n**	**%**
Depression	0.944	294	95.5	Depression	0.503	290	50.8	Depression	0.786	2308	83	Depression	0.786	1263	77.4	Obesity	0.306	1174	31.5
Anxiety	0.905	282	91.6	Anxiety	0.477	275	48.2	Anxiety	0.73	2079	74.8	Anxiety	0.73	1213	74.3	Dermatitis	0.282	1069	28.7
Obesity	0.531	167	54.2	Obesity	0.291	166	29.1	Dermatitis	0.243	659	23.7	Obesity	0.53	890	54.5	Asthma	0.136	519	13.9
Dermatitis	0.523	164	53.2	Dermatitis	0.252	145	25.4	Obesity	0.216	571	20.5	Dermatitis	0.51	856	52.5	Ch. Rhinitis	0.115	441	11.8
Asthma	0.422	134	43.5	Asthma	0.205	116	20.3	PTSD	0.134	372	13.4	Asthma	0.309	532	32.6	Ch. Sinusitis	0.086	331	8.9
SUD	0.354	115	37.3	Menorrhagia	0.101	59	10.3	Asthma	0.138	360	12.9	Migraine	0.258	446	27.3	Anxiety	0.109	331	8.9
Personality Dis.	0.324	109	35.4	Ch. Rhinitis	0.104	58	10.2	Migraine	0.103	278	10	Somat. Dis.	0.257	443	27.1	Migraine	0.088	324	8.7
PTSD	0.322	105	34.1	PTSD	0.092	50	8.8	SUD	0.104	273	9.8	Menorrhagia	0.253	434	26.6	Menorrhagia	0.074	271	7.3
GORD	0.271	89	28.9	Ch. Sinusitis	0.081	47	8.2	Menorrhagia	0.094	259	9.3	PTSD	0.246	415	25.4	Seb. Dermatitis	0.062	240	6.4
Menorrhagia	0.272	85	27.6	Migraine	0.081	46	8.1	Ch. Sinusitis	0.08	209	7.5	GORD	0.228	397	24.3	Ch. Skin Ulcer	0.062	239	6.4
Migraine	0.264	84	27.3	IBS	0.079	45	7.9	Ch. Rhinitis	0.075	197	7.1	Ch. Rhinitis	0.234	395	24.2	IBS	0.054	204	5.5
Ch. Rhinitis	0.2	64	20.8	SUD	0.065	35	6.1	IBS	0.062	168	6	Ch. Sinusitis	0.229	388	23.8	Epilepsy	0.049	187	5
Somat. Dis.	0.195	64	20.8	Epilepsy	0.063	34	6	Ensethopathy	0.055	156	5.6	IBS	0.213	364	22.3	Ensethopathy	0.046	166	4.5
IBS	0.199	63	20.5	Seb. Dermatitis	0.058	33	5.8	PCOS	0.04	116	4.2	ME	0.19	328	20.1	PTSD	0.044	158	4.2
Gastritis	0.143	45	14.6	RA	0.054	31	5.4	Dysmenorrhoea	0.037	99	3.6	SUD	0.163	275	16.9	Depression	0.060	63	1.7

sTable 4. ADHD READ Codes	
**ReadCode**	**ReadTerm**
6A61.00	Attention deficit hyperactivity disorder annual review
8BPT.00	Drug therapy ADHD (attention deficit hyperactivity disorder)
8BPT000	Stimulant drug therapy for ADHD
8BPT100	Non-stimulant drug therapy for ADHD
9Ngp.00	On drug ther ADHD (attention deficit hyperactivity disorder)
9Ngp000	On stim drug ther ADHD (attention def hyperactivity disordr)
9Ngp100	On non-stimulant drug therapy for ADHD
9Ol8.00	ADHD monitoring invitation first letter
9Ol9.00	ADHD monitoring invitation second letter
9OlA.00	ADHD monitoring invitation third letter
E2E..00	Childhood hyperkinetic syndrome
E2E..11	Overactive child syndrome
E2E0.00	Child attention deficit disorder
E2E0100	Attention deficit with hyperactivity
E2E0z00	Child attention deficit disorder NOS
E2E1.00	Hyperkinesis with developmental delay
E2E2.00	Hyperkinetic conduct disorder
E2Ey.00	Other hyperkinetic manifestation
E2Ez.00	Hyperkinetic syndrome NOS
Eu84400	[X]Overactive disorder assoc mental retard/stereotype movts
Eu90.00	[X]Hyperkinetic disorders
Eu90000	[X]Disturbance of activity and attention
Eu90011	[X]Attention deficit hyperactivity disorder
Eu90100	[X]Hyperkinetic conduct disorder
Eu90111	[X]Hyperkinetic disorder associated with conduct disorder
Eu90y00	[X]Other hyperkinetic disorders
Eu90z00	[X]Hyperkinetic disorder, unspecified
Eu90z11	[X]Hyperkinetic reaction of childhood or adolescence NOS
Eu90z12	[X]Hyperkinetic syndrome NOS
Eu9y700	[X]Attention deficit disorder
ZS91.00	Attention deficit disorder
ZS91.11	ADD - Attention deficit disorder
ZS91.12	[X]Attention deficit disorder

sTable 5. ADHD ICD-10 Codes

ICD-10 Code	Description
F90	Attention-deficit hyperactivity disorder
F90.0	Attention-deficit hyperactivity disorder, predominantly inattentive type.
F90.1	Attention-deficit hyperactivity disorder, predominantly hyperactive type.
F90.2	Attention-deficit hyperactivity disorder, combined type.
F90.8	Attention-deficit hyperactivity disorder, other type.
F90.9	Attention-deficit hyperactivity disorder, unspecified type
