# Supplementary material for: Intersecting trajectories of childhood ADHD, socioeconomic deprivation and distinct multimorbidity patterns in women
Source: Nat Ment Health. 2026 May 25;4(8):1239–47. doi: 10.1038/s44220-026-00653-1 (PMC13441990; doi:10.1038/s44220-026-00653-1)
Supplement: Supplementary file 2 [file 44220_2026_653_MOESM2_ESM.pdf]

nature portfolio

Corresponding author(s): Dr Naomi Wilson

Last updated by author(s): Oct 16, 2025

Reporting Summary

Nature Portfolio wishes to improve the reproducibility of the work that we publish. This form provides structure for consistency and transparency in reporting. For further information on Nature Portfolio policies, see our Editorial Policies and the Editorial Policy Checklist.

Please do not complete any field with "not applicable" or n/a. Refer to the help text for what text to use if an item is not relevant to your study.
For final submission: please carefully check your responses for accuracy; you will not be able to make changes later.

Statistics

For all statistical analyses, confirm that the following items are present in the figure legend, table legend, main text, or Methods section.

n/a	Confirmed	
☐	☒	The exact sample size (n) for each experimental group/condition, given as a discrete number and unit of measurement
☐	☒	A statement on whether measurements were taken from distinct samples or whether the same sample was measured repeatedly
☐	☒	The statistical test(s) used AND whether they are one- or two-sided<br>*Only common tests should be described solely by name; describe more complex techniques in the Methods section.*
☐	☒	A description of all covariates tested
☐	☒	A description of any assumptions or corrections, such as tests of normality and adjustment for multiple comparisons
☐	☒	A full description of the statistical parameters including central tendency (e.g. means) or other basic estimates (e.g. regression coefficient) AND variation (e.g. standard deviation) or associated estimates of uncertainty (e.g. confidence intervals)
☐	☒	For null hypothesis testing, the test statistic (e.g. F, t, r) with confidence intervals, effect sizes, degrees of freedom and P value noted<br>*Give P values as exact values whenever suitable.*
☒	☐	For Bayesian analysis, information on the choice of priors and Markov chain Monte Carlo settings
☒	☐	For hierarchical and complex designs, identification of the appropriate level for tests and full reporting of outcomes
☒	☐	Estimates of effect sizes (e.g. Cohen's d, Pearson's r), indicating how they were calculated

Our web collection on statistics for biologists contains articles on many of the points above.

Software and code

Policy information about availability of computer code

Data collection	Please see below.
Data analysis	Analyses took place between December 2024 and July 2025. SQL Db2 was used to interrogate data in the SAIL Databank, and all statistical analyses were conducted using R version (4.3.1). The analyses presented in this study were implemented in R (4.3.1; R Foundation for Statistical Computing, Vienna, Austria). Propensity score matching was performed using the MatchIt package (4.7.2) available from CRAN (https://cran.r-project.org/web/packages/MatchIt/index.html). Latent class analysis was conducted with the poLCA package (1.6.0.1) available from CRAN (https://cran.r-project.org/web/packages/poLCA/index.html). Standard logistic regression models were estimated using the glm() function in the base stats package of R and the Interaction Contrast Ratio was calculated as per the formula given.

For manuscripts utilizing custom algorithms or software that are central to the research but not yet described in published literature, software must be made available to editors and reviewers. We strongly encourage code deposition in a community repository (e.g. GitHub). See the Nature Portfolio guidelines for submitting code & software for further information.

Data

Policy information about availability of data

All manuscripts must include a data availability statement. This statement should provide the following information, where applicable:

- Accession codes, unique identifiers, or web links for publicly available datasets
- A description of any restrictions on data availability
- For clinical datasets or third party data, please ensure that the statement adheres to our policy

Data was collected through the Secure Anonymised Information Linkage (SAIL) Databank, a national data repository based at Swansea University, Wales, UK, which collates and links routinely collected, anonymised data from multiple health and public service sources across Wales, including primary and secondary care. Data are transferred to SAIL through secure, encrypted channels from trusted data providers, including the NHS Wales Informatics Service, Welsh Government, and other public sector organisations. A complete data availability statement is included in the final manuscript as follows: The data that support the findings of this study are available from the Secure Anonymised Information Linkage (SAIL) Databank. These data are not publicly available due to governance and privacy restrictions but can be accessed by bona fide researchers via application to the SAIL Databank's independent Information Governance Review Panel (IGRP). Access requires approval of a research proposal and must be conducted within the SAIL secure Trusted Research Environment. Further details regarding the application process are available at https://www.saildatabank.com.

Research involving human participants, their data, or biological material

Policy information about studies with human participants or human data. See also policy information about sex, gender (identity/presentation), and sexual orientation and race, ethnicity and racism.

Reporting on sex and gender	This paper focused specifically on those of female birth sex and this is made clear throughout the manuscript. The term gender has been avoided as this information was not collected and the term birth sex has been used throughout the manuscript.
Reporting on race, ethnicity, or other socially relevant groupings	Participants were additionally grouped according to socioeconomic status as determined through Welsh Index of Multiple Deprivation (WIMD), an official measure of relative area level for specific to Wales and determined by the Welsh Government. It was not possible to group participants according to race or ethnicity as this is poorly recorded within primary care records.
Population characteristics	Participants were exclusively of female birth sex and were aged between 18 and 32 at the study end point. A large proportion of those in both our exposure (39.9%) and control (28.1%) groups resided in an area of high deprivation at birth according to the Welsh Index of multiple deprivation. All other relevant population level characteristics are presented within the manuscript.
Recruitment	This is an observational cohort study which used population level data. Participants were identified/recruited from linked data hosted in the Secure Anonymised Information Linkage (SAIL) databank a population level research database thar covers approximately 86% of the Welsh population through their primary care records. Incidences of exposure, outcome and confounders were identified from a range of datasets hosted within the SAIL databank specifically: the Welsh Demographic Service Dataset; the National Community Child Health Database (NCCHD); the Welsh Longitudinal General Practice (WLGP) Database; and the Patient Episode Database for Wales (PEDW).
Ethics oversight	SAIL's Information Governance Review Panel (IGRP) granted approval to conduct this research (IGRP no. 1635).

Note that full information on the approval of the study protocol must also be provided in the manuscript.

Field-specific reporting

Please select the one below that is the best fit for your research. If you are not sure, read the appropriate sections before making your selection.

☐ Life sciences ☒ Behavioural & social sciences ☐ Ecological, evolutionary & environmental sciences

For a reference copy of the document with all sections, see nature.com/documents/nr-reporting-summary-flat.pdf

Behavioural & social sciences study design

All studies must disclose on these points even when the disclosure is negative.

Study description	This was a longitudinal cohort study used routinely collected quantitative health data, to examine the combined influence of childhood socioeconomic deprivation and childhood ADHD diagnosis on multimorbidity risk in early adulthood among females. We additionally used latent class analysis to explore multimorbidity clustering patterns among this population.
Research sample	The study sample comprised individuals included in the Secure Anonymised Information Linkage (SAIL) Databank, a population-scale repository of routinely collected health and administrative data for Wales. The cohort included females born between 1991 and 1998 and registered with a GP practice participating in SAIL before the age of 13 with linked primary and secondary care records. Demographic information, including age (mean 28.1), sex (100% female), and area-level socioeconomic deprivation, was derived from routinely recorded data within SAIL. The SAIL Databank is broadly representative of the Welsh population in terms of age, sex, and geographic distribution, supporting the generalisability of findings to this setting. The study sample was selected to enable longitudinal investigation of ADHD, socioeconomic deprivation, and multimorbidity using comprehensive, linked healthcare records.

Sampling strategy

Participants from the SAIL databank (see below) were included if they were female, born between 1991 and 1998 (inclusive), were registered with a Welsh general practice both before the age of 13 years and had not died or emigrated before the end of follow-up, and had available data on the included confounders (n=122,480). A participant flow diagram is available in Supplementary Material (Figure 1).

The study included all individuals meeting predefined inclusion criteria. No formal sample size calculation was performed, as the study utilised existing population-level data; the sample size (n = 10,263) was determined by data availability and is sufficient to support robust statistical analyses. Qualitative sampling and data saturation are not applicable.

Data collection

Data were obtained from the Secure Anonymised Information Linkage (SAIL) Databank, a national data repository based at Swansea University, Wales, UK, which contains routinely collected, anonymised electronic health and administrative records collected from multiple health and public service sources across Wales, including primary and secondary care.. Data are recorded by healthcare professionals and administrative systems as part of standard care and service provision, rather than for research purposes. Data are transferred to SAIL through secure, encrypted channels from trusted data providers, including the NHS Wales Informatics Service, Welsh Government, and other public sector organisations.No participants or researchers were directly involved in data collection, and no additional individuals were present. Blinding to experimental condition is not applicable, as this is an observational study using secondary data; however, analyses were conducted on anonymised datasets, minimising the risk of bias.

Timing

Data was collected continuously throughout the study period. For incidences of our exposure (i.e. ADHD) participants were included from birth (i.e. between 1991 and 1998 inclusive). For incidences of our outcome (i.e. multimorbidity) participants were then followed from the date of the individual's 18th birthday for all participants (i.e., regardless of timing of NDC diagnosis for each exposure group), until the earliest of the date of the outcome (multimorbidity), death, last available general practitioner registration (indicating emigration) or administrative censoring at the end of the study period (November 2023).

Data exclusions

Participants who were not registered with a Welsh GP before the age of 13 (n=229,450), who had died (n=1104) or emigrated (n=41231) before the age of 18, or who had died (n=2912) or emigrated (n=91,599) before the study end point were excluded from analyses. No further data exclusions were applied.

Non-participation

All those who met our inclusion criteria were included in the study and there were no further exclusions for non-participation.

Randomization

Participants were allocated into an experimental and control group depending on whether they had an ADHD diagnosis, recorded in their primary or secondary care records before the age of 18. While further randomization was not conducted, propensity score matching was used to minimize other differences between these groups, the methods used to achieve this are fully described within the methods section of the paper.

Reporting for specific materials, systems and methods

We require information from authors about some types of materials, experimental systems and methods used in many studies. Here, indicate whether each material, system or method listed is relevant to your study. If you are not sure if a list item applies to your research, read the appropriate section before selecting a response.

Materials & experimental systems

n/a	Involved in the study
☒	☐ Antibodies
☒	☐ Eukaryotic cell lines
☒	☐ Palaeontology and archaeology
☒	☐ Animals and other organisms
☐	☒ Clinical data
☒	☐ Dual use research of concern
☒	☐ Plants

Methods

n/a	Involved in the study
☒	☐ ChIP-seq
☒	☐ Flow cytometry
☒	☐ MRI-based neuroimaging

Clinical data

Policy information about clinical studies

All manuscripts should comply with the ICMJE guidelines for publication of clinical research and a completed CONSORT checklist must be included with all submissions.

Clinical trial registration	n/a
Study protocol	n/a
Data collection	n/a
Outcomes	n/a

Plants

Seed stocks	n/a
Novel plant genotypes	n/a
Authentication	n/a

This checklist template is licensed under a Creative Commons Attribution 4.0 International License, which permits use, sharing, adaptation, distribution and reproduction in any medium or format, as long as you give appropriate credit to the original author(s) and the source, provide a link to the Creative Commons license, and indicate if changes were made. The images or other third party material in this article are included in the article's Creative Commons license, unless indicated otherwise in a credit line to the material. If material is not included in the article's Creative Commons license and your intended use is not permitted by statutory regulation or exceeds the permitted use, you will need to obtain permission directly from the copyright holder. To view a copy of this license, visit http://creativecommons.org/licenses/by/4.0/
